# Eosinophilic annular erythema: report of four cases in adults and update of the literature^؆^

**DOI:** 10.1016/j.abd.2025.501269

**Published:** 2026-01-19

**Authors:** Emilia N. Cohen Sabban, Horacio A. Cabo, Rosario Peralta, Gabriel Salerni, Fernando Stengel, Yolanda Gilaberte, Esteban Maronna

**Affiliations:** a Department of Dermatology, Instituto de Investigaciones Médicas Alfredo Lanari, Universidad de Buenos Aires, Buenos Aires, Argentina; b Department of Dermatology, Universidad de Buenos Aires, Buenos Aires, Argentina; c Hospital Provincial del Centenario de Rosario, Universidad Nacional de Rosario, Santa Fe, Argentina; d Private Practice, Buenos Aires, Argentina; e Department of Dermatology, Instituto de Investigación Sanitaria Aragón, Hospital Universitario Miguel Servet, University of Zaragoza, Zaragoza, Spain; f Laboratory of Pathology, Sanatorio Mater Dei, Buenos Aires, Argentina

Dear Editor,

Eosinophilic annular erythema (EAE) is an uncommon, usually self-limited dermatosis, first described in infancy by Peterson in 1981.^1^ The first adult case was reported in 2000 by Kahofer et al.^2^ EAE belongs to both eosinophil-associated dermatoses and figurate erythemas as well.

The authors report four adult cases of EAE (Table 1) that were histologically proven and assessed at the same institution between 2017 and 2024. Two women and two men, aged 39 to 65 years (median age 53), presented clinically with lesions involving the trunk and extremities (Fig. 1, Fig. 2). Intense itching was the main symptom. No vesiculation, scaling, or central punctum was present. Only Case 2 resolved with post-inflammatory hyperpigmentation. Laboratory tests, including complete blood count, C-reactive protein, erythrocyte sedimentation rate, VDRL, fasting blood sugar, renal and liver function, and direct immunofluorescence, were all normal or negative. On histological examination, all cases revealed similar findings, showing no epidermal involvement. The dermis showed an intense dermal edema, vasodilation, and a moderate mixed infiltrate with abundant eosinophils in a perivascular and interstitial distribution at superficial and deep levels. No flame figures were seen (Fig. 3).

**Table 1:** Clinical data.

Case nº	Age yrs/ Gender	Concomitant disease	Type of lesions	Location	Histology	Blood test	Treatment	Duration	Relapse
1	39 Female	None	Urticarial papules and erythematous annular plaques	Trunk and extremities	Dermal edema. Superficial and deep moderate perivascular eosinophilic infiltrate.	Normal	Prednisone + anti histamines with good response	2 years	Yes
2	61 Male	None	Urticarial papules and annular plaques	Trunk and extremities	Mild spongiosis.Perivascular and interstitial mixed infiltrate with numerous eosinophils	Eosinophilia IgE 400 IU/mL	Prednisone and hydroxychloroquine were ineffective. Cyclosporine (3.5 mg/Kg/d) with good response. Post inflammatory hyperpigmentation	6 months	No
3	67 Male	Chronic eczema	Erythematous macules and plaques	Trunk and extremities	Spongiosis, dermal edema. Moderate mixed perivascular infiltrate with numerous eosinophils	Normal	Prednisone with good response	2 months	No
		Epilepsy							
		Hypothyroidism							
4	45 Female	Atopic dermatitis	Annular erythematous plaques	Trunk and abdomen	Moderate perivascular and interstitial mixed infiltrate with numerous eosinophils	Normal	Prednisone and antihistamines	After 6-months still continues	Yes
		Allergic rhinitis							
		ANA+

**Fig. 1:**
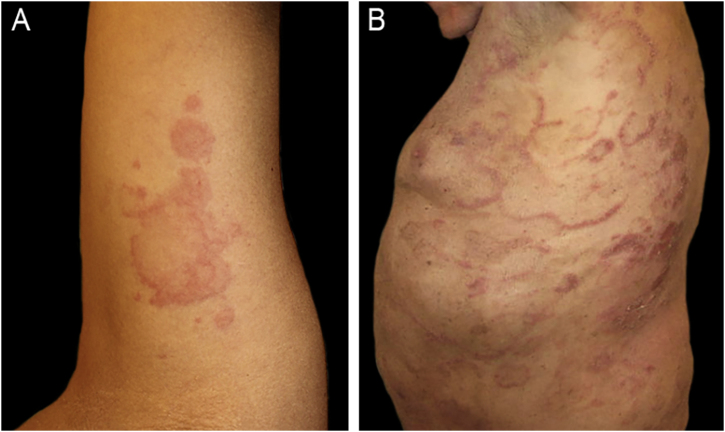
(A) Case 1: Annular plaques with erythematous borders on the right armpit and upper extremity. (B) Case 3: Erythematous macules and plaques with the typical figurate erythema aspect, on the trunk.

**Fig. 2:**
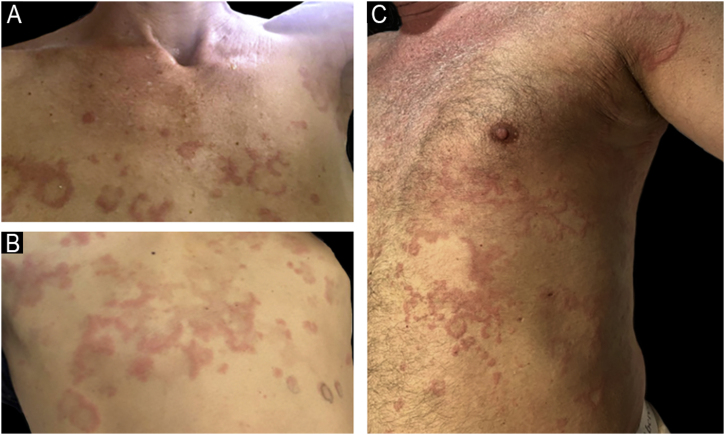
(A and B) Case 4: Annular plaques with central healing and erythematous borders on the trunk. (C) Case 2: Urticarial papules and erythematous plaques on the trunk and extremities.

**Fig. 3:**
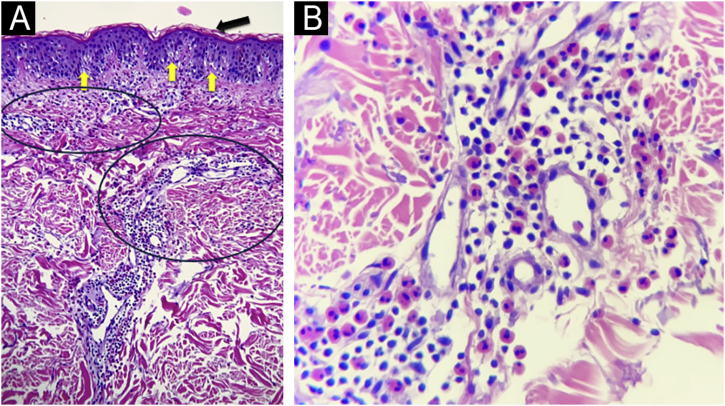
(A) Hematoxilyn & eosin, 100×. Epidermis with orthokeratosis (black arrow) and mild spongiosis (yellow arrows). Dermis with moderate inflammatory infiltrates of perivascular and interstitial disposition. (circles) (B) Hematoxilyn & eosin, 400 ×. A close up of the dermal infiltrates on a perivascular and interstitial disposition made up of lymphocytes and numerous eosinophils.

## Case 1

A healthy 39-year-old woman with no relevant past medical history presented with a five-month history of an intermittent skin eruption consisting of urticarial papules and annular plaques with erythematous borders, that enlarged centrifugally and each one disappeared without a trace, lasting up to 48 hours. There was no history of arthropod bites, exposure to pets, recent travel, or use of any medications. A skin biopsy confirmed the diagnosis of EAE. The patient received topical and oral steroids – prednisone 40 mg/od, plus a combination of three antihistamines. Prednisone was slowly tapered down to 4 mg/d over a month. The skin lesions faded gradually, and the pruritus score decreased from 10/10 to 6/10. Prednisone was stopped upon achieving complete remission. She suffered similar outbreaks over two years and finally went into remission.

## Case 2

**Fig. 4:**
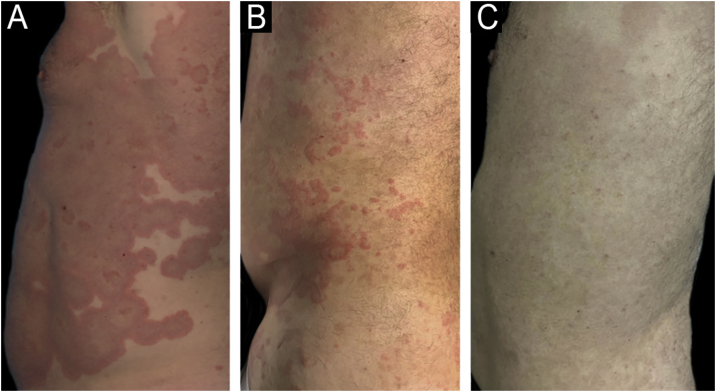
Case 2 Pre- and post-treatment. (A) Baseline (cyclosporine 3.5 mg/k/day). (B) 9 days of treatment. (C) Complete remission at 4-weeks of treatment.

A 61-year-old man with no previous medical history presented with an acute, recurrent, intensely itchy skin rash on the trunk and extremities that affected his quality of life. Physical examination revealed widespread involvement sparing the face, genitalia, oral mucosa, scalp, palms, and soles. Individual lesions had become confluent, developing into large erythematous polycyclic configurations. Laboratory tests revealed eosinophilia of 18%, and an elevated IgE 400 Iu/mL. The patient failed to respond to topical corticosteroids and high-dose antihistamines. Topical and oral prednisone ‒ 40 mg/day followed, with symptomatic relief but incomplete control of lesions. Hydroxychloroquine 200 mg/day was subsequently added with similar results. Finally, oral cyclosporine (3.5 mg/k/day) monotherapy was started, and after two weeks, the majority of the lesions resolved, with significant central post-inflammatory hyperpigmentation. Complete remission of skin lesions was achieved at 4-weeks (Fig. 4), and cyclosporine was gradually tapered and discontinued six months after initiation. At one-year follow-up, the patient has remained relapse-free.

## Case 3

A 67-year-old man suffering from chronic eczema for many years presented with a pruritic eruption of erythematous macules and plaques. He had been receiving valproic acid for epilepsy for the past 20-years, lipid-lowering therapy for the past 7-years, and levothyroxine for hypothyroidism. Skin biopsy confirmed the diagnosis. The patient was started on prednisone ‒ 40 mg/day over 40-days with a good response, tapering to 40/20 mg alternate days, then 20 mg/d over two more months, until he was free of lesions.

## Case 4

A 45-year-old healthy woman presented with a 4-months history of an intermittent pruritic rash on her trunk and abdomen. As a possible trigger factor, the patient mentioned that the skin rash started after a bee sting during her holidays. There was no history of exposure to pets or use of any medications. Except for her atopic dermatitis, recurrent allergic rhinitis, and a positive Antinuclear Antibody (ANA) at a 1:320 dilution with a fine nuclear speckled pattern (AC-4), her personal medical history and family history were otherwise normal. The patient received oral prednisone 8 mg od and a combination of two second-generation antihistamines as maintenance therapy. This combination achieved pruritus control with lesional improvement. However, relapses occurred upon withdrawal of therapy. The authors have failed to achieve disease control to date.

EAE is an acute, benign, and intensely pruritic dermatosis characterized by recurrent annular, arciform, figurate, or polycyclic erythematous and edematous plaques with a centrifugal growth pattern. The lesions usually arise on the trunk and extremities and most individual lesions tend to disappear within 36–48 hours. The absence of vesiculation and residual scaling distinguishes it from other conditions. At least 82 adult cases have been reported in the literature, including atypical variants with vesiculobullous features or palmoplantar localization.^3^, ^4^ Although self-limited, relapses are characteristic of the disease. Regarding the two patients who experienced several relapses during a 2-year follow-up, the authors found no changes in their laboratory tests or other possible explanations for this course.

The pathogenesis remains unclear. Current hypotheses suggest an Interleukin-5 (IL-5) mediated eosinophil recruitment in response to unknown triggers, possibly allergic stimulus or insect bites, as suspected in Case 4. Although eosinophilia is occasionally reported, it is not a common finding.^5^

Histopathology typically shows dermal involvement with a perivascular and interstitial infiltrate with numerous eosinophils and some lymphocytes without granulomas or vasculitis. Flame figures, characteristic of wells syndrome (WS), are generally absent. Pigmentary incontinence and basal melanosis may lead to post-inflammatory hyperpigmentation, as seen in Case 2.^5^

Differential diagnoses (Table 2) include other annular dermatoses, such as generalized granuloma annulare, erythema annulare centrifugum, subacute cutaneous lupus erythematosus, erythema multiforme, erythema gyratum repens, and erythema migrans. Eosinophilic disorders to consider include WS, bullous pemphigoid, parasitic infections, arthropod bites, and eosinophilic vasculitis.^1^

**Table 2:** Differential diagnoses of EAE.

Dermatosis	Clinical description	Histologic features
Generalized Granuloma Annulare	May occur in infancy and adulthood. Asymptomatic or occasionally pruritic, non-scaly erythematous papules and plaques with different morphology, annular, arciform, polycyclic. The etiology is unclear. It may be associated with Diabetes mellitus, thyroid diseases, infections (hepatitis B/C virus, HIV), malignancies, etc.	Pattern of either palisading granuloma with focal degeneration of collagen bundles surrounded by lymphocytes, histiocytes, and giant cells, or interstitial distribution of the infiltrate, in the upper and mid-dermis. Abundant mucin deposition
Erythema Annulare Centrifugum	Usually begins in middle age with erythematous papules and plaques that enlarge centrifugally to form rings and arcs with central hypopigmentation. On the inner part of the advancing edge, a fine collarette of scales is described. Each lesion lasts days to months. Face, trunk, extremities. Occasionally, it accompanies an underlying disease (infections, malignancies, etc.)	The epidermis often shows areas of mild spongiosis surmounted by focal parakeratosis. Dense perivascular lymphohistiocytic infiltrate in the superficial and deep dermis without eosinophils
Subacute lupus	Usually in adults. It presents with erythematous papulosquamous or annular plaques on photo-exposed areas. ANA + and Ro+	Interface dermatitis, edema, perivascular, peri-adnexal lymphocytic infiltrate. Positive Direct Immunofluorescence (DIF)
Erythema gyratum repens	Persistent concentric, arcuate, or polycyclic, scaling plaques, almost always associated with an underlying malignant neoplasm of the breast, lung, stomach, bladder, prostate.	Histologically, it shows both, epidermal (hyper and parakeratosis, focal spongiosis), and a dermal component composed by mild perivascular mononuclear infiltrate
Tinea corporis	Annular erythematous plaques with peripheral leading scale and central healing	Direct potassium hydroxide microscopy (KOH) and culture lead to the diagnoses of superficial fungal infection
Wells syndrome	Clinically, it´s characterized by prodromal burning, painful edema, and peripheral induration. The classical description is a cellulitis-like plaque, but then, its clinical polymorphism with erythematous annular and figurate lesions have been described. The lesions usually resolved within a few weeks leaving a slate-grey morphea-like induration. The recurrent course and spontaneous healing after months or years, are features that both entities share.	Prominent papillary dermal edema, and interstitial infiltrate mainly eosinophils, are diffusely distributed, and can be localized at the superficial, mid and/or deep dermis and extending into the subcutis. Typical flame figures consisting of degenerated collagen fibers, due to deposits of the major basic protein of the eosinophil.
Erythema Gyratum Atrophicans	Occurs in newborns as a generalized eruption of erythematous plaques evolving to atrophy and hypopigmentation.	Epidermal atrophy, dermal edema, and a mononuclear cell infiltrate are present. DIF reveals granular deposits of IgG, C3, and C4 at the dermoepidermal junction and around the superficial capillaries
Erythema Multiforme	May occur at any age presenting as symmetrically distributed papules, annular plaques and target-like lesions typically fixed for a minimum of seven days.	Interface dermatitis characterized by epidermal basal cell damage, which may be manifested by cell death and/or basal vacuolar change, and a lichenoid distribution of inflammatory cells obscuring the dermoepidermal interface
Erythema Chronicum Migrans	There is often a history of an arthropod bite and a resulting central punctum. The non-scaly, usually unique plaque, expands centrifugally as a blue-red urticarial ring over several weeks or months.	Mononuclear peri-adnexal and perivascular dermal infiltrate is present.
Urticaria	Edematous papules that rapidly became large and irregular plaques intensely pruritic. Each lesion lasts no more than 6 -hs and they arise on different locations.	Edema and sparse perivascular inflammatory infiltrate in the upper dermis
Annular Erythema of infancy	Annular and figurate pruritic lesions with early age of onset (under 1-year of age) and spontaneous resolution on the trunk and extremities. No prodromes. Sparing the face, genitalia, palms and soles.	Perivascular mononuclear infiltrate with few eosinophils; negative DIF; lack of mucin, “flame figures”, vasculitis or granulomas
	No Eosinophilia in the majority of cases.	
	Recurrent, chronic, with outbreaks.	

There is an ongoing debate regarding whether EAE is a separate entity or a variant of WS. Some authors propose that EAE represents a chronic, treatment-resistant form of WS with higher relapse rates. However, the absence of flame figures, lack of blood eosinophilia, and distinct clinical features support the classification of EAE as an independent condition.^6^

Associations between EAE and systemic diseases such as autoimmune thyroiditis (Case 3), diabetes mellitus, and systemic lupus erythematosus have been described. El-Khalawany et al. observed that managing systemic comorbidities may contribute to longer remission periods and reduced recurrence.^7^

Treatment should be individualized based on symptom severity, lesion extent, and associated conditions. Therapeutic options include topical and systemic corticosteroids (0.5–1 mg/kg/day), oral minocycline or doxycycline, hydroxychloroquine (200–400 mg/day), dapsone (50–100 mg/day), low-dose cyclosporine, methotrexate, and antihistamines, alone or in combination. Clinical improvement usually occurred within 2–6 weeks. However, relapses after drug discontinuation are frequent. One case in the literature reported the successful use of narrowband UVB in a corticosteroid and hydroxychloroquine-resistant patient, achieving long-term remission.^8^, ^9^

Recent reports have also described successful treatment of refractory cases with biologics such as dupilumab, mepolizumab, benralizumab, and the JAK inhibitor baricitinib, though these remain off-label and are based on isolated cases.^10^

In conclusion, the authors propose that EAE is a distinct clinical entity, characterized by annular, pruritic, polycyclic plaques. This presentation differs from the more edematous, cellulitis-like lesions of WS, which commonly exhibit peripheral eosinophilia and flame figures on histology. Skin biopsy remains essential for diagnosis. Although therapeutic responses are usually favorable, relapses are common. Further studies are needed to clarify its pathogenesis, classification, and optimal long-term management.

## ORCID ID

Emilia N. Cohen Sabban: 0000-0002-5941-7439

Horacio A. Cabo: 0000-0002-8563-7013

Gabriel Salerni: 0000-0001-6386-4402

Fernando Stengel: 0009-0000-7681-2548

Yolanda Gilaberte: 0000-0001-8034-3617

Esteban Maronna: 0000-0002-5144-896X

## Financial support

None declared.

## Authors' contributions

Emilia N. Cohen Sabban: Data collection, analysis and interpretation; preparation and writing of the manuscript; approval of the final version of the manuscript.

Horacio A. Cabo: Intellectual participation in propaedeutic and/or therapeutic management of studied cases and approval of the final version of the manuscript.

Rosario Peralta: Manuscript critical review, critical literature review and approval of the final version of the manuscript.

Gabriel Salerni: Effective participation in research orientation and approval of the final version of the manuscript.

Fernando Stengel: Critical literature review and approval of the final version of the manuscript.

Yolanda Gilaberte: Preparation and writing of the manuscript and approval of the final version of the manuscript.

Esteban Maronna: Data collection, analysis and interpretation and approval of the final version of the manuscript.

## Research data availability

The entire dataset supporting the results of this study was published in this article.

## Conflicts of interest

None declared.
